# Application of a Glucose Dehydrogenase-Fused with Zinc Finger Protein to Label DNA Aptamers for the Electrochemical Detection of VEGF

**DOI:** 10.3390/s20143878

**Published:** 2020-07-11

**Authors:** Jinhee Lee, Atsuro Tatsumi, Kaori Tsukakoshi, Ellie D. Wilson, Koichi Abe, Koji Sode, Kazunori Ikebukuro

**Affiliations:** 1 Joint Department of Biomedical Engineering, The University of North Carolina at Chapel Hill and North Carolina State University, Chapel Hill, NC 27599, USA; jh.lee@unc.edu (J.L.); elliedw@email.unc.edu (E.D.W.); ksode@email.unc.edu (K.S.); 2 Department of Biotechnology and Life Science, Graduate School of Engineering, Tokyo University of Agriculture and Technology, 2-24-16 Naka-cho, Koganei, Tokyo 184-8588, Japan; atsuro0916tatsumi@gmail.com (A.T.); k-tsuka@cc.tuat.ac.jp (K.T.); abe79kou@gmail.com (K.A.)

**Keywords:** aptamer, labeling, enzyme, zinc finger protein, glucose dehydrogenase, electrochemical sensor, vascular endothelial growth factor

## Abstract

Aptamer-based electrochemical sensors have gained attention in the context of developing a diagnostic biomarker detection method because of their rapid response, miniaturization ability, stability, and design flexibility. In such detection systems, enzymes are often used as labels to amplify the electrochemical signal. We have focused on glucose dehydrogenase (GDH) as a labeling enzyme for electrochemical detection owing to its high enzymatic activity, availability, and well-established electrochemical principle and platform. However, it is difficult and laborious to obtain one to one labeling of a GDH-aptamer complex with conventional chemical conjugation methods. In this study, we used GDH that was genetically fused to a DNA binding protein, i.e., zinc finger protein (ZF). Fused GDH can be attached to an aptamer spontaneously and site specifically in a buffer by exploiting the sequence-specific binding ability of ZF. Using such a fusion protein, we labeled a vascular endothelial growth factor (VEGF)-binding aptamer with GDH and detected the target electrochemically. As a result, upon the addition of glucose, the GDH labeled on the aptamer generated an amperometric signal, and the current response increased dependent on the VEGF concentration. Eventually, the developed electrochemical sensor proved to detect VEGF levels as low as 105 pM, thereby successfully demonstrating the concept of using ZF-fused GDH to enzymatically label aptamers.

## 1. Introduction

Aptamers are DNA or RNA ligands that recognize a specific target molecule with comparable affinity and specificity to antibodies [1,2]. The utilization of aptamers in biosensing has several advantages over antibodies owing to their small size, high thermal stability, and ease of chemical modification. Accordingly, aptamers have become a major target recognition element in a variety of biosensing principles, including fluorescence, electrochemical, and colorimetric methods. In particular, since our first report on aptamer-based electrochemical sensors [3], many electrochemical aptamer sensors have been described for point of care testing purposes, taking advantage of rapid response times, ease of miniaturization, and cost-effectiveness owing to their established platform over other electrochemical detection principles.

To construct such electrochemical sensors, enzymes are attractive and often used as labeling molecules for high-sensitivity detection owing to their signal amplification ability. In reported electrochemical aptamer sensors, alkaline phosphatase and horse radish peroxidase are the most common enzymatic labels [4]. To label aptamers, avidin is typically conjugated to these enzymes, and this avidin-enzyme conjugate interacts with a biotinylated aptamer to form a chimeric aptamer-enzyme molecule. Historically, alkaline phosphatase and horse radish peroxidase are both difficult enzymes to produce recombinantly via prokaryotic host cells, such as *Escherichia coli*. Therefore, the preparation of avidin-enzyme complexes synthesized via chemical conjugation is favorable. However, the procedure to chemically conjugate and purify avidin and enzymes of interest before their use is not only laborious, but it can result in heterogenous molecules due to random conjugation [5] as well as the loss of protein function [6].

Alternatively, we have focused on glucose dehydrogenase (GDH) as a labeling enzyme for electrochemical aptamer sensors [3,7,8]. As GDH is the enzyme employed in the representative miniaturized electrochemical biosensor, the glucose sensor for the self-monitoring of blood glucose, well-established electrochemical principles and platforms are available. Importantly, GDH can be produced via prokaryotes while retaining a high specific activity, allowing for easy engineering of the enzyme [9]. Therefore, GDH would serve as an attractive labeling enzyme to engineer and perform simple and site-specific labeling for electrochemical aptamer sensors.

With regard to site-specific modifications, several methods are reported utilizing unnatural amino acids [6], intein [10], enzyme-catalyzed tags [11], self-labeling enzymes [12], or DNA-templated conjugation [13,14]. However, additional materials other than the DNA and protein are required to perform these conjugation methods. Kobatake and Gordon’s group have developed a unique strategy for DNA–protein crosslinking through a DNA-binding enzyme-mediated covalent bond formation [15,16,17]. However, the method still suffered from low bioactivity of the conjugated enzyme. Inspired by our previous study [18], we have focused on the zinc finger protein (ZF), a monomeric double-stranded DNA-binding protein, to create a GDH-labelled aptamer. ZF is a DNA-binding protein which is capable of recognizing and binding to a specific sequence of double-stranded DNA [19,20]. On account of its small molecular weight, various molecules, including enzymes and fluorescent proteins, have been genetically fused to ZF for the purpose of gene editing and DNA detection, and are efficiently produced using prokaryotic cells without compromising its original function [21,22,23,24,25]. Previously, we have reported the use of ZF-fused GDH for the detection of PCR products, and successfully detected target DNA quantitatively [25]. This study demonstrated that the binding of ZF toward DNA happens spontaneously in a neutral buffer solution in a site-specific manner in vitro. Therefore, we assumed the aptamer labeling of GDH would occur by simply mixing the aptamer with ZF-fused GDH.

To this end, we present a novel GDH labeling method using ZF-fused GDH for aptamers. As a proof of concept, we have utilized vascular endothelial growth factor (VEGF) as a model target. VEGF plays an important role in the formation of new blood vessels of tissues [26]. As anti-VEGF pharmaceuticals are being used in tumor and age-related macular degeneration therapies, the detection of VEGF is important to predict the effectiveness of these treatments [27,28,29]. Moreover, VEGF is suggested as a biomarker for the early diagnosis of several diseases, such as lymphangioleiomyomatosis [30], major depressive disorder [31], and diabetic nephropathy [32]. Therefore, the rapid and sensitive detection of VEGF levels is crucial for clinical purposes. Here, we present the construction and results of an electrochemical sensor based on a GDH-labeled aptamer for VEGF detection with a lower detection limit of 105 pM.

## 2. Materials and Methods

### 2.1. Chemicals and Materials

All oligonucleotides were purchased from Eurofins Genomics (Luxembourg, Luxembourg). The sequences are indicated in the Supplementary Materials Table S1.

Phenazine methosulfate (PMS), 2,6-dichlorophenolindophenol (DCIP), d(+)-glucose, sodium chloride, 2-amino-2-hydroxymethyl-propane-1,3-diol (Tris), glycerol, and Tween-20 were purchased from Kanto Chemical Co. Inc. (Tokyo, Japan). Kanamycin sulfate and d-desthiobiotin were purchased from Sigma-Aldrich Co. LLC (St. Louis, MO, USA). A self-assembled monolayer (SAM) forming thiol reagent (dithiobis(succinimidyl undecanoate)) was purchased from Dojindo Laboratories Co., Ltd. (Kumamoto, Japan). LB broth, the bacterial host strain *Escherichia coli* BL21(DE3), and the expression vector pET30c(+) were purchased form Merck KGaA (Darmstadt, Germany). Anti-VEGF antibodies (MAB293 and BAF293) were purchased from R&D Systems, Inc. (Minneapolis, MN, USA). Gold and platinum wires were purchased from TANAKA Kikinzoku (Tokyo, Japan). A silver/silver chloride (3M NaCl) reference electrode RE-1B (Ag/AgCl) was purchased from BAS Inc. (Tokyo, Japan).

### 2.2. Investigation of Aptamer and Antibody Combination for the Sandwich Assay

Either biotinylated VEGF aptamers (30 pmol/well) or anti-VEGF antibody; BAF293 (20 pg/well) was diluted by TBS (10 mM Tris-HCl and 100 mM NaCl; pH 7.0) and immobilized on a streptavidin-coated micro-titer plate (Nunc, Rochester, NY, USA) by incubating for 1 h at 25 °C. Subsequently, the wells were washed with TBS-T (10 mM Tris-HCl, 100 mM NaCl and 0.05% Tween-20; pH 7.0) and blocked by blocking buffer (TBS-T containing 4% skim milk). After blocking, 0 or 100 nM VEGF was added, incubated for 1 h, and washed again by TBS-T. Next, the 300 nM of the labeling VEGF aptamer harboring the Zif268 binding site was added and incubated. Finally, 100 nM ZF-GDH was added to each well and incubated. After a final washing by TBS-T, the residual GDH activity was measured using PMS and DCIP. For this measurement, 100 µL of assay buffer (TBS containing 0.06 mM DCIP, 0.6 mM PMS, and 100 mM glucose) was added to each well and the absorbance change at 595 nm was measured using a plate reader (Wallac 1420 ARVO MX, Perkin-Elmer, Waltham, MA, USA).

### 2.3. Investigation of Binding Specificity of ZF-GDH Towards Its Target Sequence

Biotinylated VEGF-binding aptamer (2G19) or Zif268 recognition sequence-inserted 2G19 (2G19-Z) was first heat-treated at 95 °C for 10 min then gradually cooled down to 25 °C in TBS to let them fold into a stable structure. Then, 100 µL of the folded oligonucleotide solution was added onto a streptavidin coated micro-titer plate (Nunc, Rochester, NY, USA) at a concentration of 1 µM. After immobilization, each well was washed with TBS-T three times and blocked with a blocking buffer. Finally, 100 µL of 100 nM ZF–GDH solution was added and incubated for 1 h at 25 °C. After washing with TBS-T, the binding of ZF–GDH to each oligonucleotide was detected by measuring the residual GDH activity using PMS and DCIP using a plate reader (Wallac 1420 ARVO MX, Perkin-Elmer).

### 2.4. Investigation of VEGF Concentration Dependency on Plate Using Antibody and Aptamer

To select the pair of ligands for the sandwich binding assay, either biotinylated aptamer (30 pmol/well) or the anti-VEGF antibody BAF293 (20 pg/well) was immobilized on a streptavidin coated clear micro-titer plate by incubating for 1 h at 25 °C. Subsequently, the wells were washed with TBS-T and blocked using a blocking buffer. After blocking, 0 or 100 nM VEGF was added and incubated for 1 h, followed again by washing with TBS-T. Next, 100 µL of 300 nM 2G19-Z or V7t1 aptamer was added and incubated. Finally, 100 nM ZF–GDH was added to each well and incubated. After a final wash by TBS-T, the residual GDH activity was measured as described in Section 2.2. For the investigation of VEGF concentration dependency, 0–100 nM VEGF solution was added on the antibody-immobilized micro-titer plate and incubated for 1 h at room temperature. After washing, the captured VEGF was detected by GDH labeled 2G19-Z.

### 2.5. Preparation of Antibody-Immobilized Gold Electrode

For the formation of the self-assembled monolayer (SAM) on a gold wire electrode, the wire was cleaned by piranha solution and immersed in ethanol containing 1 mM thiol reagent (dithiobis(succinimidy undecanoate)) overnight at 25 °C. Next, the SAM-modified electrode was immersed into HEPES buffer (pH 7.0) containing 0.15 mg/mL streptavidin and incubated overnight at 4 °C. Followingly, the electrode was incubated for 30 min in HEPES buffer containing 5 µg/mL biotinylated anti-VEGF antibody (BAF293). Finally, the *N*-hydroxysuccinimide (NHS) ester was blocked through incubation of 1 M Tris-HCl (pH 8.0) for 15 min.

### 2.6. Investigation of the Non-Specific Adsorption on a Gold Electrode

A bare gold wire electrode, SAM-modified electrode, or antibody immobilized electrode was incubated with 100 nM ZF-GDH in TBS for 30 min. After washing, the residual GDH activity was electrochemically measured using chronoamperometry. The prepared gold electrode (working electrode) was immersed in 10 mL TBS containing 1 mM electron mediator (m-PMS: 1-methoxy-5-methylphenazinium methylsulfate) with Ag/AgCl and Pt wire as reference and counter electrodes, respectively. After applying a potential of 0.1 V (vs. Ag/AgCl) in a VersaSTAT4 potentiostat (Princeton Applied Research, Princeton, NJ, USA), the background response current was measured as the background in the absence of glucose. The applied potential was determined by considering the standard redox potential of mPMS (+0.063 V) [33], and we confirmed the potential was able to effectively oxidize mPMS in our previously reported GDH-based amperometric aptamer sensor [3,7,8]. Then, 25 s after application of the potential, glucose was added at a final concentration of 100 mM and the response current was measured for 100 s. The delta current was determined by subtracting the background current before the addition of glucose from the plateau signals after the addition of glucose.

### 2.7. Electrochemical Detection of VEGF Using GDH-Labeled Aptamer

The BAF293-immobilized gold wire electrode was blocked with 4% skim milk in TBS-T and incubated in TBS containing 0.25–25 nM of VEGF for 30 min at room temperature. Following washing with TBS-T, the electrode was incubated with 300 nM of 2G10-Z for 30 min and was subsequently incubated with 100 nM of ZF–GDH for 30 min again. After a final wash, residual GDH activity was detected via the same amperometric method described in the Section 2.6 with 1 mM m-PMS and 100 mM glucose utilizing VersaSTAT4 potentiostat (Princeton Applied Research, Princeton, NJ, USA). The calibration of VEGF was obtained by plotting the delta current against VEGF concentration. As a negative control, bovine serum albumin (BSA) was incubated with the electrode instead of VEGF, and after incubation of the aptamer and ZF-GDH, the residual GDH activity was measured by the chronoamperometry with an applied potential of 0.1 V (vs. Ag/AgCl).

## 3. Results

### 3.1. GDH Labeling of VEGF-Binding Aptamer Using ZF-GDH and Characterization of the Labeled Aptamer

To construct the fusion of GDH and ZF, we chose GDH derived from *Aspergillus flavus* (AfGDH) [34] and a mouse-derived ZF (Zif268) [35]. AfGDH is a monomeric flavin adenine dinucleotide-dependent GDH which has high catalytic activity of several hundred Unit/mg protein. Zif268 is a very well-characterized C_2_H_2_-type ZF which binds to its target double-stranded DNA (forward strand: 5′-GCGTGGGCG-3′ and reverse strand: 5′-CGCCCACGC-3′) in a one-to-one manner with high sequence specificity and binding affinity [35]. We confirmed the fusion protein maintained both the target double-stranded DNA binding ability of Zif268 [25] and the enzymatic activity of AfGDH. Remarkably, the enzymatic activity was the almost same level as that of original AfGDH (Supplementary Materials Figure S1). This is due to the small molecular weight of ZF (<10 kDa), one of the advantages of ZF as a fusion partner.

For electrochemical detection of VEGF, we selected a sandwich manner of detection. Therefore, first, the best combination of ligands was investigated on a sandwich plate assay. For this comparison, we chose two types of VEGF binding aptamers (Supplementary Materials Table S1). Both aptamers have a single binding region against VEGF with a nano-molar order *K*_d_ value, but fold into different unique structures. Moreover, 2G19 aptamer is predicted to form a stem loop structure [36], whereas V7t1 aptamer was revealed to form a G-quadruplex (G4) structure [37,38]. To label them by ZF-GDH, 2G19 and V7t1 were fused to a ZF recognition site and referred to as 2G19-Z and V7t1-Z, respectively (Supplementary Materials Figure S2). As the capture ligand, we immobilized either biotinylated VEGF aptamer or anti-VEGF antibody on a streptavidin-coated micro-titer plate. After the addition of 100 nM VEGF, GDH-labeled 2G19-Z or V7t1-Z was further added and washed. Eventually, the residual GDH activity was detected with a colorimetric redox dye.

**Figure 1:**
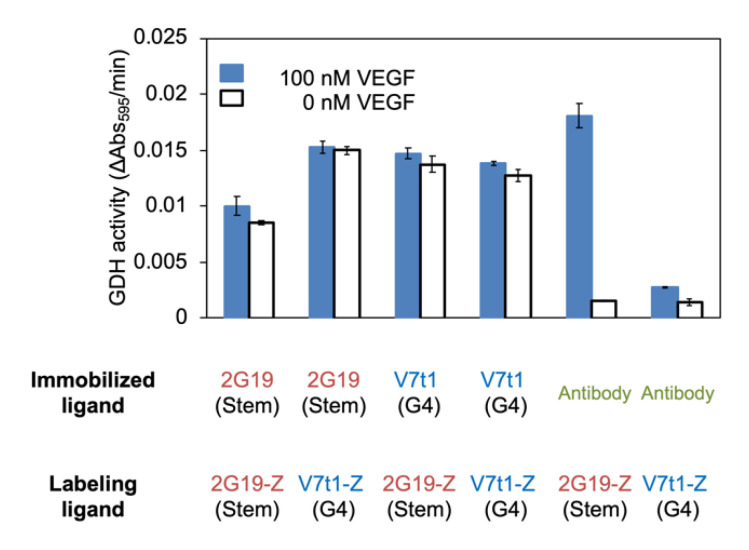
Investigation of aptamer and antibody combinations for the sandwich assay. To capture VEGF, aptamers (2G19 or V7t1) or anti-VEGF antibody (BAF293) was immobilized on a streptavidin-coated microtiter plate. As the detection ligand, ZF-GDH-labeled aptamers (2G19-Z or V7t1-Z) were used. Residual GDH activity on the plate was detected with colorimetric redox dye. Error bars indicate the standard deviation (*n* = 3).

When we used the anti-VEGF antibody as the capture ligand, the GDH-labeled aptamers showed a signal increase in the presence of VEGF (Figure 1). This indicated that both aptamers were correctly labeled by ZF-GDH and can be used to detect VEGF regardless of folding structure. Whereas, all of the aptamer-aptamer pairings showed a higher background signal without the addition of VEGF compared to that of antibody-aptamer pairs (Figure 1). Considering the low background signal with the antibody–aptamer pairs, this higher background signal would be due to the direct interaction between the capture and labeling aptamers rather than the non-specific adsorption of ZF-GDH. Therefore, we chose the anti-VEGF antibody and 2G19 aptamer pair to construct the VEGF sensor, as this combination provided the highest signal with the lowest background noise.

The specific binding of ZF-GDH to the inserted Zif268-binding site in 2G19 was further investigated on a micro-titer plate. Here, we immobilized either the original 2G19 or Zif268 binding site inserted 2G19 (2G19-Z) using biotin-avidin interaction and allowed ZF-GDH to bind to the DNA. After washing, the residual GDH activity was measured with a colorimetric redox dye. We observed a significantly higher signal from the wells immobilized with 2G19-Z (Supplementary Materials Figure S3). This confirmed that ZF-GDH bound to the specific sequence inserted in the 2G19-Z aptamer. In other words, ZF-GDH labeled the aptamer with GDH.

Finally, we investigated VEGF concentration dependency using anti-VEGF antibody and GDH-labeled 2G19-Z on a micro-titer plate. Various concentrations of VEGF solution were added to the plate, and the VEGF captured by the antibody was detected using the GDH-labeled aptamer. When GDH activity was measured, signal increase was dependent on VEGF concentration, while there was no signal increase with the negative control protein, bovine serum albumin (BSA) (Supplementary Materials Figure S4). Therefore, as we expected, ZF-GDH functioned to label the aptamer, which showed a specific binding against VEGF.

### 3.2. Construction of Electrochemical Detection System Using GDH-Labeled 2G19-Z to Detect VEGF

To construct the electrochemical VEGF detection system using the GDH-labeled aptamer, we modified the SAM on the gold wire electrode (approximate working surface area: 16 mm^2^) using a decyl alkyl chain harboring a thiol group and *N*-hydroxysuccinimide (NHS) group to immobilize anti-VEGF antibody and to reduce the non-specific adsorption of ZF-GDH. The non-specific adsorption of ZF-GDH on the SAM-modified electrode was investigated by measuring the residual GDH activity after the electrode was immersed in the ZF-GDH solution. GDH activity was measured using the chronoamperometry in the presence of 1 mM m-PMS and 100 mM glucose with an applied potential of 0.1 V (vs. Ag/AgCl). When the bare gold electrode was incubated in the ZF-GDH solution, a high response current was observed even after washing with a buffer; however, the current was reduced with the SAM- and antibody-modified electrode (Supplementary Materials Figure S5). Therefore, the immobilization of this SAM on the electrode efficiently reduced non-specific adsorption.

**Figure 2:**
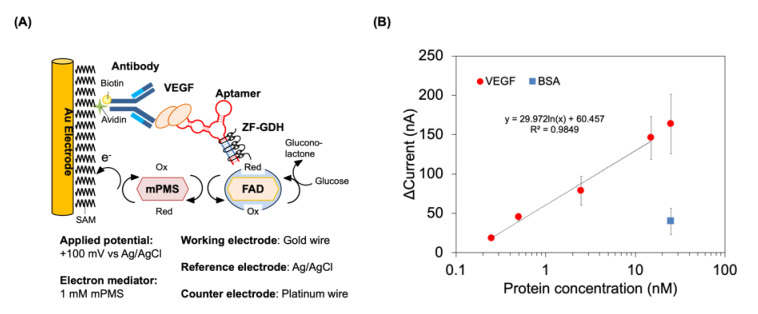
Electrochemical detection of VEGF. (**A**) Schematic diagram of the electrochemical detection of VEGF. (**B**) Electrochemical detection of VEGF by using ZF-GDH-labelled aptamer. The GDH activity was measured by chronoamperometry in the presence of 1 mM mPMS and 100 mM glucose with an applied potential of 0.1 V (vs. Ag/AgCl). The delta current is defined as the subtraction of background current before addition of glucose from the plateau signals after the addition of glucose. Error bars indicate standard deviations (*n* = 3).

To immobilize the antibody on the SAM-modified electrode, we used biotin-avidin interactions to control the orientation of the anti-VEGF antibody. First, streptavidin was immobilized on the SAM-modified electrode via amine coupling, and then biotinylated anti-VEGF antibody was immobilized on a streptavidin-modified electrode. After antibody immobilization, the surface was blocked by skim milk and used to detect VEGF. For this detection, we incubated the electrode with 0.25–25 nM of VEGF and after washing, the captured VEGF was labeled by 2G19-Z and ZF-GDH (Figure 2A). In this principle, the amount of GDH on the electrode would be dependent on the amount of the captured VEGF. Upon addition of glucose, GDH obtains an electron and the cofactor (flavin adenine dinucleotide) is reduced. Subsequently, the cofactor is then re-oxidized by the free electron mediator existing in solution, mPMS. Eventually, the reduced mPMS is oxidized by the electrode to generate the response current for the chronoamperometry measurement. Therefore, as the VEGF concentration increases, more reduced mediator is generated, and a higher response current is observed.

As a result of detection, we observed a concentration-dependent signal increase with a linear relationship between the current responses and the logarithmic values of VEGF concentration ranging from 0.25–15 nM (Figure 2B). The limit of detection was calculated to be 105 pM according to the method of M_b_ + 3 SD (where M_b_ and SD are the mean value and standard deviation of the blank, respectively). However, the addition of the negative control, BSA instead of VEGF, gave a higher current value than the blank and was comparable to the current value obtained with 0.5 nM VEGF. This indicated that further optimization of the electrode surface modification is necessary.

Although the sensitivity can be improved as the estimated VEGF concentration in a sample would be sub-pico molar, these results showed that the aptamer was correctly labeled with GDH and generated an electrochemical signal. Most importantly, the spontaneous binding of the ZF to the binding sequence inserted into the aptamer enabled GDH labeling of the aptamer by merely mixing the solution.

## 4. Discussion

For the simple fabrication and construction of an enzyme-based amperometric aptamer sensor, we focused on GDH in terms of its facile recombinant production and engineering. Specifically, we circumvented GDH labeling via chemical conjugation by using ZF-fused GDH. As we expected, the ZF-GDH fusion protein was expressed as soluble protein in *E. coli*-based recombinant production system. In contrast to chemical conjugation, the fusion of ZF had little effect on GDH function, as ZF-fused GDH maintained more than 90% of original GDH activity (Supplementary Materials Figure S1). With chemical modification, surface lysine residues are commonly targeted, and the random modification of these residues can decrease the solubility and stability of protein. As we fused ZF genetically to the N-terminus of GDH, the surface lysine residues remained intact. Additionally, considering the fused position of ZF, which is separate from the active site, and the small molecular weight (<10 kDa) of ZF, the fusion of ZF did not alter protein folding of the GDH nor block the substrate access.

When we labeled two different VEGF-binding aptamers using ZF-GDH and performed sandwich detection of VEGF, both aptamers showed higher GDH activity in the presence of VEGF. Although their folding structures are different, both aptamers were labeled by GDH and served as a detection ligand for VEGF. Therefore, this suggests that this GDH labeling method can be generalized for different aptamers than those used in this study. In the sandwich assay, the G-quadruplex forming V7t1 aptamer showed lower signal intensity than 2G19 aptamer in spite of its better *K*_d_ value (1.4 nM vs. 52 nM). Therefore, the sensitivity depends on the paring of antibody and aptamer, not just on the aptamer’s affinity.

We successfully detected VEGF electrochemically utilizing the aptamer labeled by ZF-GDH with a lower detection limit of 105 pM and a detection range of 250 pM to 15 nM. To date, several electrochemical aptamer sensors are reported for VEGF detection (Supplementary Materials Table S2). Some of these sensors utilize labeling enzymes, such as alkaline phosphatase or GDH, however they also require chemical conjugation of avidin. We were able to bypass this laborious process by using ZF-GDH to label the aptamer. Besides the simplicity of labeling, another advantage of the present system in comparison to other reported sensors is its detection principle. Namely, the system is an amperometric sensor based on GDH activity, and this principle is adopted in the commercially available glucose sensors. Therefore, we can construct this sensing system with already established platforms. On the other hand, the sensor showed a signal increase with BSA, a non-target molecule. This current increase can be attributed to the direct interaction of ZF-GDH with the non-specifically adsorbed BSA on the electrode surface. As seen in the result of the sandwich plate assay (Supplementary Materials Figure S4), once the surface was sufficiently blocked, the GDH-labeled 2G19-Z did not show a signal increase with the addition of BSA. Therefore, further optimization of the SAM composition and its density, as well as the conditions of blocking and washing would prove this signal increase with BSA.

Compared to previously reported GDH activity-based VEGF sensors, our sensor demonstrated a tenfold improvement in (Supplementary Materials Table S2). However, our sensor sensitivity was moderate in comparison to those reported for electrochemical sensors, as some demonstrated femto-molar levels of sensitivity. Indeed, the constructed sensor requires improvement considering the sub-pico molar concentration of VEGF in a clinical sample. To increase the signal, we can first consider fusing multiple GDH enzymes to ZF to obtain higher GDH activity. Secondly, we can increase the affinity of ZF to DNA by increasing the number of ZF motifs, as well as alter these motifs to recognize longer sequences. In this study, we used Zif268 which recognizes 9 bp and has a *K*_d_ with nanomolar affinity, but by fusing an additional ZF motif, we can improve the *K*_d_ to a picomolar level [39].

## 5. Conclusions

In this study, we employed GDH as a labeling enzyme for an amperometric aptamer sensor and developed a GDH labeling method for a VEGF-binding aptamer using ZF-fused GDH. Since GDH can be recombinantly produced and easily engineered, the connecter, ZF was genetically fused to GDH, eliminating the need for chemical conjugation methods to prepare ZF-fused GDH. The small molecular size of ZF (<10 kDa) contributed to the maintenance of GDH activity even after the fusion of ZF. Furthermore, the fusion protein enabled simple and easy labeling of the aptamer without laborious steps, such as purification and dialysis, to obtain the aptamer–GDH complex. Eventually, we could detect 105 pM VEGF in the GDH activity-based electrochemical detection system. Since the target specificity of ZF is designable, this GDH labeling method has the potential to be a universal labeling method to construct electrochemical aptamer sensors.

## Supplementary Materials

The following are available online at https://www.mdpi.com/1424-8220/20/14/3878/s1, Supplemental Table S1: Sequences of oligonucleotides, Supplemental Table S2: Comparison of electrochemical VEGF aptamer sensors, Preparation of GDH-fused ZF, Figure S1: Kinetic parameters of purified AfGDH wild type and ZF-GDH, Figure S2: Predicted secondary structure of VEGF-binding aptamers after fusing ZF binding site, Figure S3: Binding specificity of ZF-GDH, Figure S4: VEGF concentration dependency on the microtiter plate, Figure S5: Effect of SAM modification on non-specific absorption of ZF-GDH.
