# Supplementary material for: Application of a Glucose Dehydrogenase-Fused with Zinc Finger Protein to Label DNA Aptamers for the Electrochemical Detection of VEGF
Source: Sensors (Basel). 2020 Jul 11;20(14):3878. doi: 10.3390/s20143878 (PMC7411789; doi:10.3390/s20143878)
Supplement: Supplementary file 1 [file sensors-20-03878-s001.pdf]

sensors

Supplemental information

Application of a Glucose Dehydrogenase-Fused with Zinc Finger Protein to Label DNA Aptamers for the Electrochemical Detection of VEGF

Jinhee Lee [1,†], Atsuro Tatsumi [2,†], Kaori Tsukakoshi [2,†], Ellie D. Wilson [1], Koichi Abe [2], Koji Sode [1] and Kazunori Ikebukuro [2,*]

[1] Joint Department of Biomedical Engineering, The University of North Carolina at Chapel Hill and North Carolina State University, Chapel Hill, NC 27599, USA; jh.lee@unc.edu (J.L.); elliedw@email.unc.edu (E.D.W.); ksode@email.unc.edu (K.S.)

[2] Department of Biotechnology and Life Science, Graduate School of Engineering, Tokyo University of Agriculture and Technology, 2-24-16 Naka-cho, Koganei, Tokyo 184-8588, Japan; atsuro0916tatsumi@gmail.com (A.T.); k-tsuka@cc.tuat.ac.jp (K.T.); abe79kou@gmail.com (K.A.)

* Correspondence: ikebu@cc.tuat.ac.jp; Tel.: +81-42-388-7030

† These authors contributed equally to this work.

Table S1. Sequences of oligonucleotides.

Name	Sequence (5′ to 3′)
2G19	CTGGCCAGGTACCAAAAGATGATCTTGGGCCCGTCCGAATGGTGGGTGTTCTGGCCAG
2G19-Z	GATGCGTGGGCGAGGTACCAAAAGATGATCTTGGGCCCGTCCGAATGGTGGGTGTTCTCGCCC ACGCATC
V7t1	TGTGGGGGTGGACGGGCCGGGTAGA
V7t1-Z	TATGCGTGGGCGACTTTTGTCGCCCACGCATATGTGGGGGTGGACGGGCCGGGTAGA

Table S2. Comparison of electrochemical VEGF aptamer sensors.

Year	Principle	LOD	Detection Range	Labels	Chemical Conjugation	Reference
2009	Field-effect transistor-based assay	104 pM	-	-	-	[1]
2010		400 fM	-	-	-	[2]
2012		100 fM	-	-	-	[3]
2012	Differential pulse voltammetry	21.4 fM	26 fM to 26 pM	Streptavidin-alkaline phosphatase	Yes	[4]
2014		1 nM	-	Hemin	-	[5]
2015		0.32 pM	1 pM to 120 pM	Methylene blue	Yes	[6]
2015		30 nM	-	Streptavidin-alkaline phosphatase	Yes	[7]
2017		0.7 pM	2.5 pM to 320 pM	Thionine	Yes	[8]
2016	Alternating current voltammetry	6.2 nM	-	Methylene blue	Yes	[9]

2017	Square wave voltammetry	3.9 pM	3.9 pM to 2.6 nM	Methylene blue	Yes	[10]
2019		0.3 fM	1 fM to 120 pM	Gold/graphene quantum dot	Yes	[11]
2015	Eectrochemical impedance spectroscopy (EIS)	0.48 pM	2.5 pM to 250 pM	-	-	[6]
2015		26 fM	260 fM to 7.8 pM	-	-	[12]
2019		176 fM	-	-	-	[13]
2015	Non-Faradaic EIS	10.4 pM	130 fM to 26 pM	Magnetic beads	Yes	[14]
2018	Photoelectrochemical assay	30 fM	100 fM to 10 nM	Methylene blue	Yes	[15]
2019		3 fM	10 fM to 100 nM	Porous carbon spheres	Yes	[16]
2012	Amperometry	50 nM	-	GDH	Yes	[17]
2012		15 nM	-	GDH	Yes	[18]
2016		4.6 pM	6 pM to 20 pM	Platinum	Yes	[19]
-		105 pM	250 pM to 25 nM	GDH	-	This work

Preparation of GDH-fused ZF

An *Aspergillus flavus*-derived dinucleotide-dependent GDH structural gene was synthesized by GenScript Inc. (Piscataway, NJ, USA), with codons optimized for expression in *E. coli.* After the removal of the sequence encoding the secretion signal peptide, the gene fragment was fused at the N-terminal of Zif268. Additionally, Strep-tag II was added to the N-terminus of this Zif268 for affinity purification. The constructed gene fragment was cloned into the *Nde*I and *Hin*dIII sites of the expression vector pET-30c(+) (Merck KGaA, Darmstadt, Germany). For recombinant production, *E. coli* BL21(DE3) (Merck KGaA, Darmstadt, Germany) was transformed using the expression vector and used for culture. The transformant was cultured at 20 °C in an autoinduction medium (LB medium containing 50 μg/mL kanamycin, 0.5% (w/v) glycerol, 0.2% (w/v) α-lactose, 0.05% (w/v) D(+)-glucose, 25 mM $(NH_4)_2SO_4$, 50 mM KH_2PO_4, 50 mM Na_2HPO_4, and 1 mM $MgSO_4$). After 24 h of incubation, the cells were lysed in a French press, then the Strep-tagged ZF–GDH was purified using Streptactin super flow plus (QIAGEN, Hilden, Germany).

Purified GDH-fused ZF was assayed for GDH activity by spectroscopic assay. The enzyme solution was mixed with reaction buffer (10 mM Tris-HCl, 100 mM NaCl (pH 7.0), 0.06 mM 2,6-dichlorophenol indophenol (DCIP), and 0.6 mM phenazine methosulfate (PMS)). Then, the reaction was initiated by adding D(+)-glucose, and the rate of the reaction was determined by monitoring the decrease in absorbance of DCIP at 600 nm using a Bio Spec UV 1200 instrument (Shimadzu Corporation, Kyoto, Japan). The amount of an enzyme that reduced 1 μmol DCIP is defined as 1 unit (U), and 16.3 mM^{-1} cm^{-1} was used as the molar absorption coefficient of DCIP at pH 7.0. Based on the obtained enzyme kinetics curve, the maximum rate of reaction toward glucose (V_{max}) and Michaelis constant (K_m) was determined.

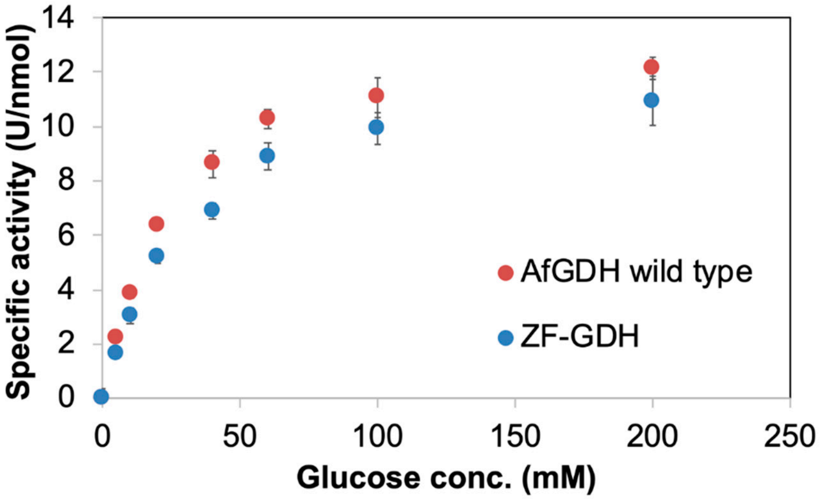

	V_{max} (U/nmol)	K_m (mM)
AfGDH wild type	15	29
ZF-GDH	14	37

Figure S1. Kinetic parameters of purified AfGDH wild type and ZF-GDH.

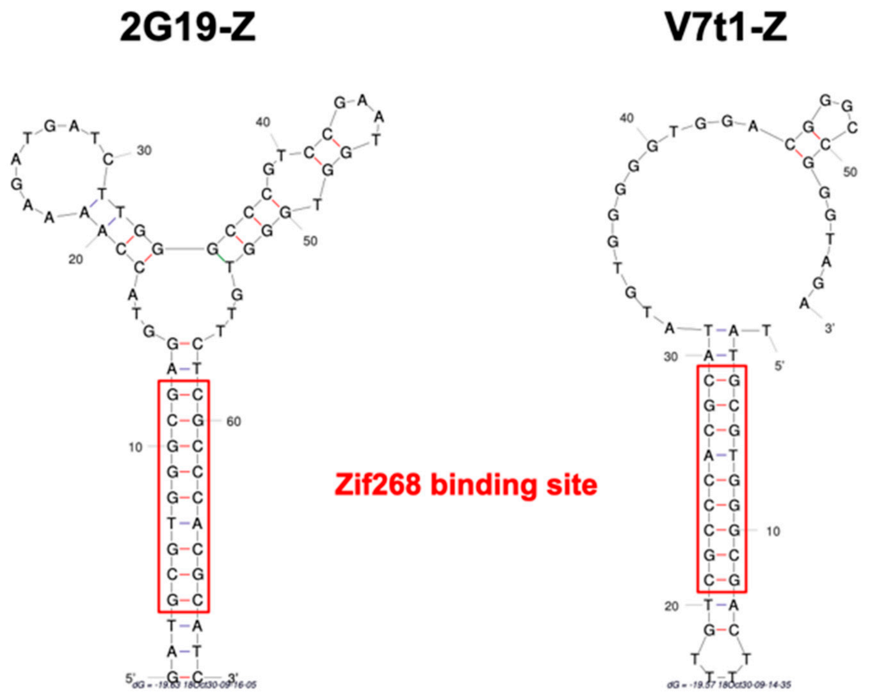

Figure S2. Predicted secondary structure of VEGF-binding aptamers after fusing ZF binding site. The prediction was performed by m-fold web server (http://unafold.rna.albany.edu/?q=mfold).

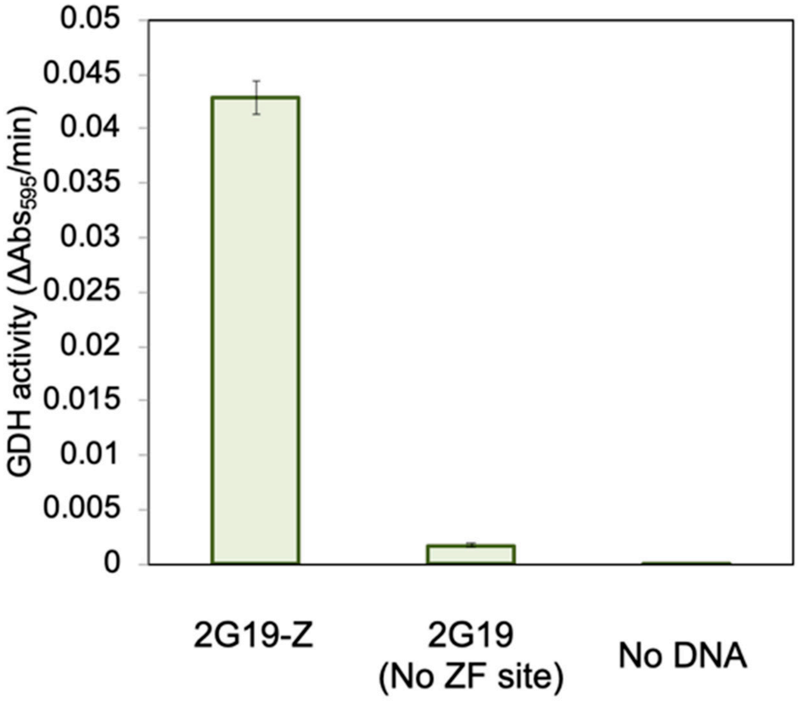

Figure S3. Binding specificity of ZF-GDH. ZF binding site harboring aptamer (2G19-Z) or the original VEGF-binding aptamer (2G19) were immobilized on streptavidin micro titer plate, and detected by ZF-GDH. Error bars indicate standard deviations (n = 3).

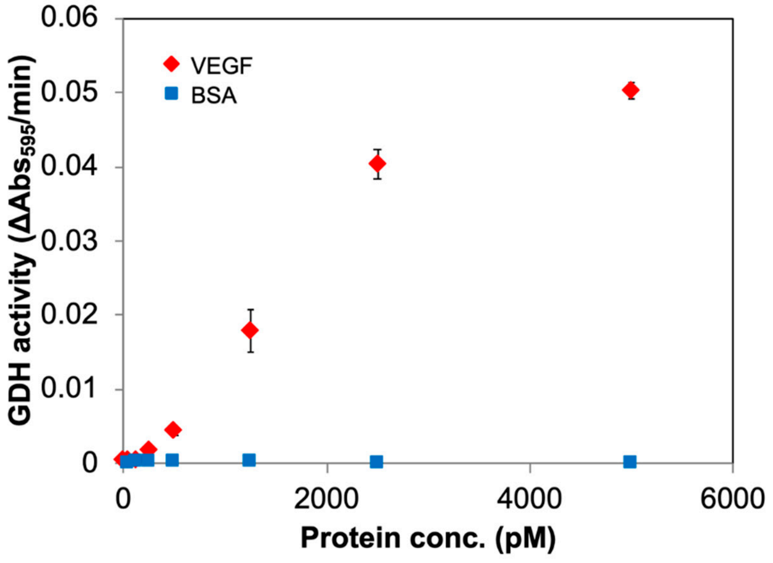

Figure S4. VEGF concentration dependency on the microtiter plate. Various concentrations of VEGF solution were incubated in the antibody-immobilized microtiter plate and washed. The captured VEGF was detected by GDH-labeled 2G19-Z. The GDH activity was detected with the redox dyes, PMS and DCIP and the calibration curve was obtained. The lower detection limit was 250 pM (S/N ≥ 3).

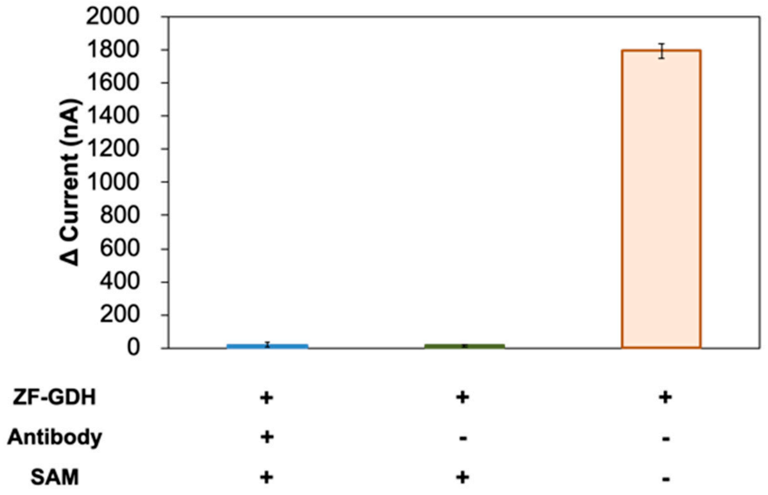

Figure S5. Effect of self-assembled monolayer (SAM) modification effect on non-specific absorption of ZF-GDH. Bare gold wire electrode, SAM modified, or SAM-Antibody modified electrode were incubated with 100 nM ZF-GDH and washed by TBS. The residual GDH activity on the electrode was measured by chronoamperometry in the presence of 1 mM electron mediator (m-PMS: 1-Methoxy-5-methylphenazinium methylsulfate) and 100 mM glucose. The response current was measured by using platinum counter electrode at a potential of 0.1 V (vs. Ag/AgCl) using VersaSTAT4 potentiostat from Princeton Applied Research (Princeton, NJ, USA). The delta current is defined the subtraction of background current before addition of glucose from the plateau signals after the addition of glucose. Here, "+" indicates the incubation of each molecule with the gold wire electrode, and "−" indicates the lack of incubation. Error bars indicate standard deviations (n = 3).

References

1. Lee, H.S.; Kim, K.S.; Kim, C.J.; Hahn, S.K.; Jo, M.H. Electrical detection of VEGFs for cancer diagnoses using anti-vascular endotherial growth factor aptamer-modified Si nanowire FETs. *Biosens. Bioelectron.* **2009,** *24*, 1801–1805, doi:10.1016/j.bios.2008.08.036.
2. Kwon, O.S.; Park, S.J.; Jang, J. A high-performance VEGF aptamer functionalized polypyrrole nanotube biosensor. *Biomaterials* **2010,** *31*, 4740–4747, doi:10.1016/j.biomaterials.2010.02.040.
3. Kwon, O.S.; Park, S.J.; Hong, J.Y.; Han, A.R.; Lee, J.S.; Lee, J.S.; Oh, J.H.; Jang, J. Flexible FET-type VEGF aptasensor based on nitrogen-doped graphene converted from conducting polymer. *ACS Nano* **2012,** *6*, 1486–1493, doi:10.1021/nn204395n.
4. Cheng, W.; Ding, S.; Li, Q.; Yu, T.; Yin, Y.; Ju, H.; Ren, G. A simple electrochemical aptasensor for ultrasensitive protein detection using cyclic target-induced primer extension. *Biosens. Bioelectron.* **2012,** *36*, 12–17, doi:10.1016/j.bios.2012.03.032.
5. Lv, Z.; Wang, K.; Zhang, X. A new electrochemical aptasensor for the analysis of the vascular endothelial growth factor. *J. Immunoassay Immunochem.* **2014,** *35*, 233–240, doi:10.1080/15321819.2013.841194.
6. Shamsipur, M.; Farzin, L.; Amouzadeh Tabrizi, M.; Molaabasi, F. Highly sensitive label free electrochemical detection of VGEF165 tumor marker based on "signal off" and "signal on" strategies using an anti-VEGF165 aptamer immobilized BSA-gold nanoclusters/ionic liquid/glassy carbon electrode. *Biosens. Bioelectron.* **2015,** *74*, 369–375, doi:10.1016/j.bios.2015.06.079.
7. Ravalli, A.; Rivas, L.; De la Escosura-Muniz, A.; Pons, J.; Merkoci, A.; Marrazza, G. A DNA Aptasensor for Electrochemical Detection of Vascular Endothelial Growth Factor. *J. Nanosci. Nanotechnol.* **2015,** *15*, 3411–3416, doi:10.1166/jnn.2015.10037.
8. Amouzadeh Tabrizi, M.; Shamsipur, M.; Saber, R.; Sarkar, S. Simultaneous determination of CYC and VEGF165 tumor markers based on immobilization of flavin adenine dinucleotide and thionine as probes on reduced graphene oxide-poly(amidoamine)/gold nanocomposite modified dual working screen-printed electrode. *Sens. Actuators B Chem.* **2017,** *240*, 1174–1181, doi:10.1016/j.snb.2016.09.108.
9. Feng, L.; Lyu, Z.; Offenhäusser, A.; Mayer, D. Electrochemically triggered aptamer immobilization via click reaction for vascular endothelial growth factor detection. *Eng. Life Sci.* **2016,** *16*, 550–559, doi:10.1002/elsc.201600068.

10. Crulhas, B.P.; Karpik, A.E.; Delella, F.K.; Castro, G.R.; Pedrosa, V.A. Electrochemical aptamer-based biosensor developed to monitor PSA and VEGF released by prostate cancer cells. *Anal. Bioanal. Chem.* **2017,** *409*, 6771–6780, doi:10.1007/s00216-017-0630-1.
11. Hongxia, C.; Zaijun, L.; Ruiyi, L.; Guangli, W.; Zhiguo, G. Molecular machine and gold/graphene quantum dot hybrid based dual amplification strategy for voltammetric detection of VEGF165. *Microchim. Acta* **2019,** *186*, 242, doi:10.1007/s00604-019-3336-6.
12. .Amouzadeh Tabrizi, M.; Shamsipur, M.; Farzin, L. A high sensitive electrochemical aptasensor for the determination of VEGF (165) in serum of lung cancer patient. *Biosens. Bioelectron.* **2015,** *74*, 764–769, doi:10.1016/j.bios.2015.07.032.
13. Wang, H.; Ma, Y.; Guo, C.; Yang, Y.; Peng, Z.; Liu, Z.; Zhang, Z. Templated seed-mediated derived Au nanoarchitectures embedded with nanochitosan: Sensitive electrochemical aptasensor for vascular endothelial growth factor and living MCF-7 cell detection. *Appl. Surf. Sci.* **2019,** *481*, 505–514, doi:10.1016/j.apsusc.2019.03.148.
14. Qureshi, A.; Gurbuz, Y.; Niazi, J.H. Capacitive aptamer–antibody based sandwich assay for the detection of VEGF cancer biomarker in serum. *Sens. Actuators B Chem.* **2015,** *209*, 645–651, doi:10.1016/j.snb.2014.12.040
15. Da, H.; Liu, H.; Zheng, Y.; Yuan, R.; Chai, Y. A highly sensitive VEGF165 photoelectrochemical biosensor fabricated by assembly of aptamer bridged DNA networks. *Biosens. Bioelectron.* **2018,** *101*, 213–218, doi:10.1016/j.bios.2017.10.032.
16. Liu, Y.L.; Da, H.M.; Chai, Y.Q.; Yuan, R.; Liu, H.Y. Photoelectrochemical aptamer-based sensing of the vascular endothelial growth factor by adjusting the light harvesting efficiency of g-C3N4 via porous carbon spheres. *Mikrochim. Acta* **2019,** *186*, 275, doi:10.1007/s00604-019-3393-x.
17. Abe, K.; Hasegawa, H.; Ikebukuro, K. Electrochemical Detection of Vascular Endothelial Growth Factor by an Aptamer-Based Bound/Free Separation System. *Electrochemistry* **2012,** *80*, 348–352, doi:10.5796/electrochemistry.80.348.
18. Nonaka, Y.; Abe, K.; Ikebukuro, K. Electrochemical Detection of Vascular Endothelial Growth Factor with Aptamer Sandwich. *Electrochemistry* **2012,** *80*, 363–366, doi:10.5796/electrochemistry.80.363.
19. Fu, X.-M.; Liu, Z.-J.; Cai, S.-X.; Zhao, Y.-P.; Wu, D.-Z.; Li, C.-Y.; Chen, J.-H. Electrochemical aptasensor for the detection of vascular endothelial growth factor (VEGF) based on DNA-templated Ag/Pt bimetallic nanoclusters. *Chinese Chem. Lett.* **2016,** *27*, 920–926, doi:10.1016/j.cclet.2016.04.014.

© 2020 by the authors. Licensee MDPI, Basel, Switzerland. This article is an open access article distributed under the terms and conditions of the Creative Commons Attribution (CC BY) license (http://creativecommons.org/licenses/by/4.0/).
